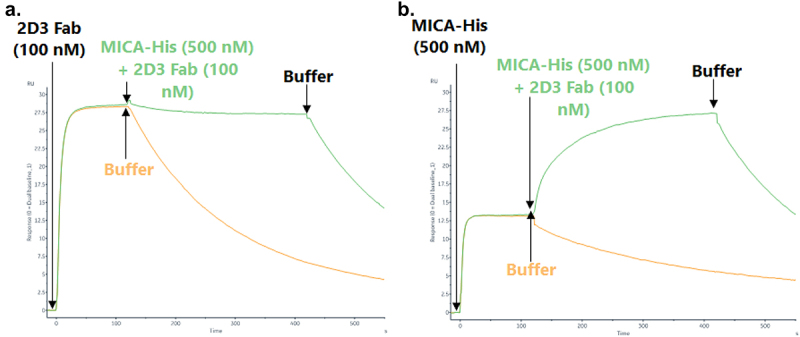# Correction

**DOI:** 10.1080/19420862.2025.2458393

**Published:** 2025-01-29

**Article title**: Agonistic anti-NKG2D antibody structure reveals unique stoichiometry and epitope compared to natural ligands

**Authors**: Fallon, D., Huang, C.-H., Ma, J., Morgan, C., & Zhou, Z. S.

**Journal**: *mAbs*

**DOI**: https://doi.org/10.1080/19420862.2024.2433121

The author has noticed an error in Figure 5. When re-submitting the figures in a higher quality format, as per the production editor’s request, the two sensorgrams were mistakenly swapped (Figure 5A and 5B). The author has updated the figure and requests that Figure 5 be updated with the revised version provided below, as it more accurately reflects the original intentions.

**Figure 5:**